# Clinical Presentations of Lumbar Disc Degeneration and Lumbosacral Nerve Lesions

**DOI:** 10.1155/2020/2919625

**Published:** 2020-08-29

**Authors:** Worku Abie Liyew

**Affiliations:** Biomedical Science Department, School of Medicine, Debre Markos University, Debre Markos, Ethiopia

## Abstract

Lumbar disc degeneration is defined as the wear and tear of lumbar intervertebral disc, and it is mainly occurring at L3-L4 and L4-S1 vertebrae. Lumbar disc degeneration may lead to disc bulging, osteophytes, loss of disc space, and compression and irritation of the adjacent nerve root. Clinical presentations associated with lumbar disc degeneration and lumbosacral nerve lesion are discogenic pain, radical pain, muscular weakness, and cutaneous. Discogenic pain is usually felt in the lumbar region, or sometimes, it may feel in the buttocks, down to the upper thighs, and it is typically presented with sudden forced flexion and/or rotational moment. Radical pain, muscular weakness, and sensory defects associated with lumbosacral nerve lesions are distributed on lower extremities, the buttock, lower abdomen, and groin region. A lumbosacral plexus lesion presents different symptoms in the territories of the lumbar and sacral nerves. Patients with lumbar plexus lesion clinically present with weakness of hip flexion, knee extension, thigh adduction, and sensory loss in the lower abdomen, inguinal region, and over the entire medial, lateral, and anterior surfaces of the thigh and the medial lower leg, while sacral plexus lesion presents clinical symptoms at nerve fibers destined for the sciatic nerve, common peroneal nerve, and pudendal nerve. Weakness of ankle inversion, plantar flexion, and foot drop are the main clinical manifestations of the sacral plexus lesion area. Numbness and decreased sensation are also present along the anterolateral calf and dorsum of the foot. On examination, foot eversion is usually stronger than foot dorsiflexion. The patients may also present with pain and difficulty of bowel movements, sexual dysfunction assessments, and loss of cutaneous sensation in the areas of the anal canal, anus, labia major, labia minor, clitoris, penis, and scrotum.

## 1. Background of the Study

**Figure 1:**
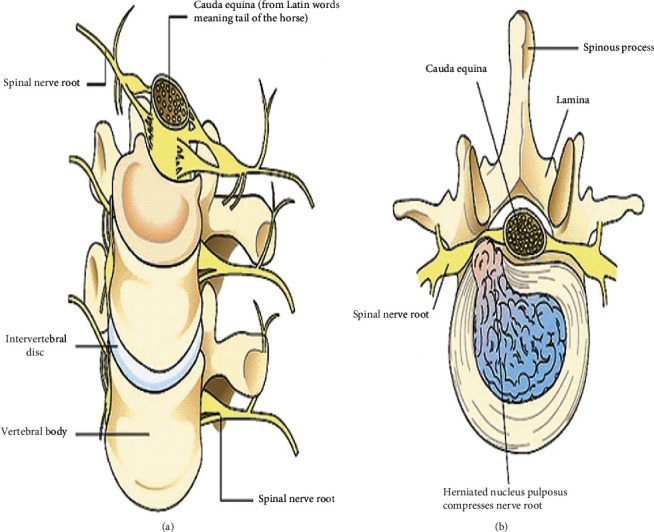
(a) Normal intervertebral disc and spinal nerve root. (b) Degenerated intervertebral disc and pinched spinal nerve root [63].

Lumbar disc degeneration is defined as the wear and tear of lumbar disc that act as a cushion for the spine. Lumbar disc degeneration can occur at any level, but mainly, it occurs on L3-L4 and L4-S1 vertebrae [1, 2]. It begins with small tears in the annulus of the disc to a decrease in the water content of the nucleus pulposus of the discs. The degenerative disc leads to disc bulging, osteophytes, disc space loss, and compression and irritation of the adjacent nerves [3]. With advanced degeneration, it loses water content and disc height (Figure 1), and it leads to segmental instability and causes degenerative spondylosis and scoliosis. The advanced degenerative changes affect disc facet joints and surrounding soft tissue and can result in canal narrowing also known as degenerative stenosis [3]. Because each lumbar disc is in direct contact with two or three pairs of dorsal roots, disc degeneration may compress the adjacent nerve root [4, 5]. This can cause the pain syndrome but, more characteristically, causes neuropathic pain and neurological symptoms and, in severe cases, dysfunction of the nerve [6, 7].

Risk factors causing lumbar disc degeneration disease and associated lumbosacral nerve compression includes advancing age, socioeconomic status [8], torsional stress [9], smoking, obesity [10–12], heavy lifting, vibration [10], trauma, immobilization [13], psychosocial factors, gender, height, hereditary, genetic factors [8, 11], and occupations like machine drivers, carpenters, and office workers [14–16]. Genetic inheritance plays a significant role in the rate of degradation. Approximately 50–70% disc degeneration is caused by an individual's genetic inheritance [17, 18]. Disc degeneration becomes prevalent and common in the individual's 40s and usually in the lower lumbar spine. Some individuals, however, can become inflicted by this disease much earlier than the norm, depending on both the severity of their genetic deficiencies and lifestyles.

Lumbar disc degeneration and associated nerve lesion account for a large amount of lost productivity in the workforce. It is the most common cause of lower back pain throughout the world [3]. Lower back pain is the single most common cause of disability at the age of 45 years and the second most common reason for primary care physician visits [2, 8]. Intervertebral degeneration and associated low back pain have a huge socioeconomic impact and place a burden on health services worldwide. People throughout the world spend more than 100 billion US dollars/year for the treatment of low back pain [2].

In lumbar disc degeneration, accurate diagnosis is difficult, treatment is controversial, and failures are common. MRI is considered to be the cornerstone and special investigation to confirm the diagnosis of LDD and associated nerve lesions. However, between 38% and 52% of asymptomatic individuals demonstrated significant lumbar disc bulging on MRI [19, 20]. Some physicians may diagnosis lumbar disc degeneration on MRI with ought to detail clinical presentations, and others may use back pain alone as a symptom of lumbar disc degeneration [21, 22]. For example, a significant imaging finding of a right disc bulge at L5/S1 in a patient with symptoms of left L4/L5 nerve root distribution is a discordant finding [23]. As a result, imaging findings may not correlate with a patient's disc problem and it results in the distrust of physicians on the part of patients and vice versa [22, 24, 25]. A basic understanding of the clinical presentations and pain distribution is important in the case of lumbar disc degeneration, and associated lumbosacral nerve lesion is important to diagnosis and disease conditions of a patient with the suspected lumbar disc degeneration. Therefore, the goal of this review is to assess all possible peripheral clinical presentations for lumbar disc degeneration and associated lumbosacral nerve lesions. The discussion of this review is limited to the peripheral clinical presentations and symptoms of lumbar disc degeneration and lumbosacral nerve lesions in the lower back region.

## 2. Anatomy of Healthy Lumbar Intervertebral Discs and Lumbosacral Nerves

In the lumbar region of the spine, there are five fibrocartilaginous lumbar intervertebral discs [26] which are named based on the vertebrae above and below them, for example, the L4-L5 disc found between L4 and L5 vertebrae. Lumbar intervertebral discs are important to transfer body weight and muscle activity arising from the upper body region to the lower body region. They also provide flexibility, extension, flexion, and torsion and provide protection to the spinal nerves, spinal cord, and the vertebrae themselves [27].

Compared to the discs of the thoracic and cervical spine, the lumbar discs are taller and wider measuring approximately 7–10 mm in thickness and 4 cm in diameter (anterior-posterior plane) [28]. The lumbar discs become shorter during the day due to the weight of the upper body, and sleeping for a minimum of 5 hours helps the discs regain their original shape [27]. The lumbar discs tend to be of greater height anteriorly than posteriorly, and this tendency is especially the greatest being the L5/S1 disc, causing the lumbar spine's natural convex curvature similar to the cervical spine [29, 30]. Morphologically, the discs are cylindrical with its shape being determined by the integrity of the annulus fibrosus [31]. Because of the mobility of the lumbar spine and the high loads applied to it, discs have a significantly higher chance of becoming damaged from bending and torsion, making it the most common spinal part for disc injury [32]. 90% of lumbar disc degeneration occurs at the L4-L5 or the L5-S1 disc space [27].

**Figure 2:**
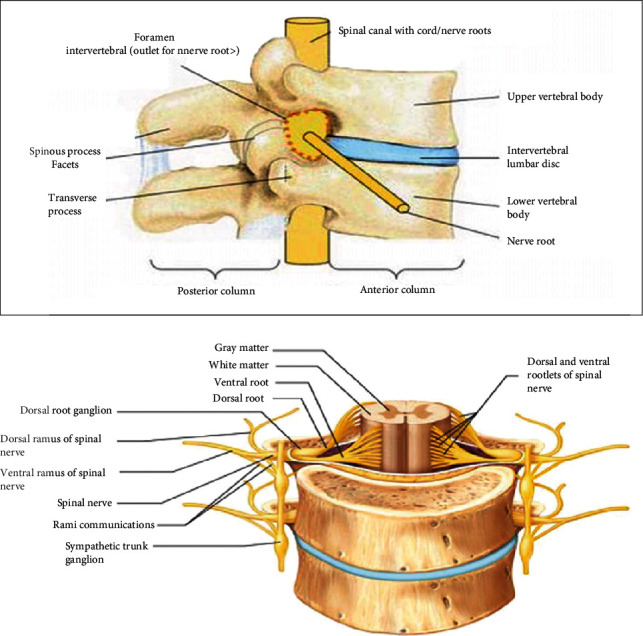
Lumbar discs and adjacent lumbar nerve roots [38, 64].

Posterior to the lumbar intervertebral disc, there are five paired anterior and posterior lumbar nerve roots (L1-L5) that exit below the corresponding lumbar vertebra through the respective foramen (Figure 2) [5]. Upon exiting the spinal column, the posterior and anterior spinal nerve roots combine around the intervertebral foramen and form five paired mixed lumbar spinal nerves. The mixed spinal nerves contain both motor and sensory nerve fibers. Mixed spinal nerves immediately divide into posterior ramus and anterior ramus. The posterior and anterior rami contain both sensory and motor nerve fibers [33]. Since most disc herniations occur posterolaterally, the root that exits the foramen below the herniated disc gets compressed. So, a disc bulge at L4/L5 will compress the L5 root, and a protrusion at L5/S1 will compress the S1 root.

**Figure 3:**
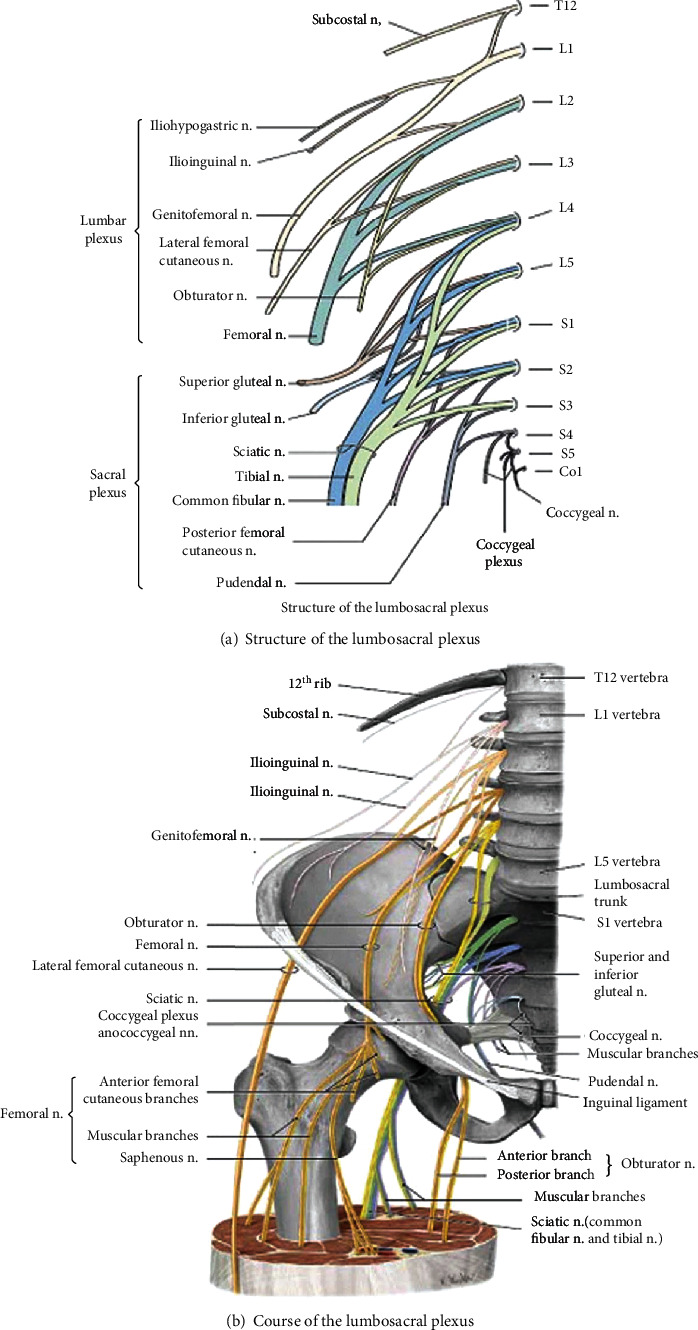
Anatomy of the lumbosacral plexus [26].

The ventral rami of the lumbar and sacral nerves (L1- S4) form the lumbosacral plexus of the body (Figure 3). Because some fibers from the lumbar plexus contribute to the sacral plexus via the lumbosacral trunk, the two plexuses are often considered together as the lumbosacral plexus. The lumbar plexus is formed by roots from L1 to L4, and the sacral plexus is by L4–S4 roots. The lumbosacral plexus gives branches that innervate structures of the lower abdomen, some pelvic genitalia, and lower limbs.

**Figure 4:**
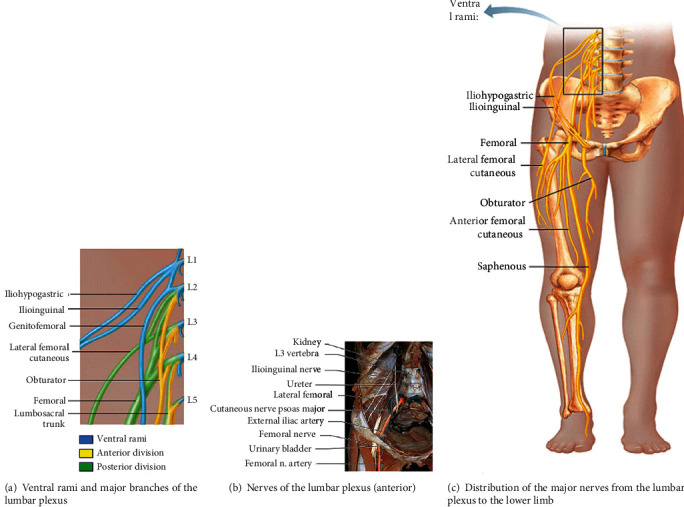
Anatomy of the lumbar plexus [38].

The lumbar plexus is located on the anterior surface of the posterior abdominal wall. The important nerves emerging from lumbar plexuses are the femoral nerve (the posterior division of the anterior primary rami of L2-L4), the obturator nerve (the anterior division of the anterior primary rami of L2-l4), lateral femoral cutaneous nerve (posterior division of the anterior rami of L2-L3), and iliohypogastric, ilioinguinal, and genitofemoral nerves, which originate mainly from L1 (Figure 4). Lumbar nerves are responsible for thigh flexion and adduction and leg extension and provide sensory innervation of the anterior and lateral thigh and medial regions of the leg [34]. The iliohypogastric, ilioinguinal, and genitofemoral nerves are important to innervate transverse and the oblique abdominal muscles [34, 35].

**Figure 5:**
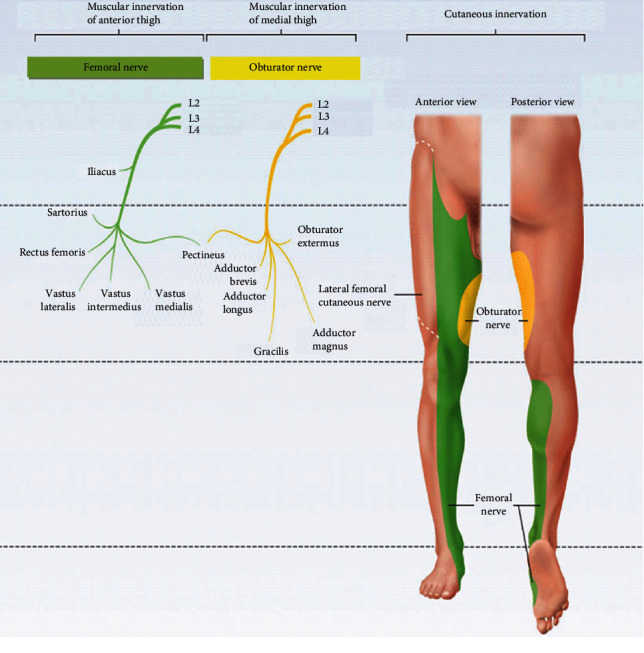
Anatomy of femoral and obturator nerves and their innervation [38].

The femoral nerve is the largest terminal branch of the lumbar plexus. It provides motor innervation to the anterior thigh muscles (quadriceps) and sensory innervation to the skin of the anterior thigh and the anteromedial aspect of the leg (Figure 5). The femoral nerve arises from the posterior cords of the lumbar plexus (L2-L4) and passes deep to the inguinal ligament. It descends vertically to the anterior thigh through the center of the femoral triangle, just lateral to the femoral artery and vein. Once it passes the inguinal ligament, it divides into deep motor branches and superficial cutaneous branches. The superficial branch divides into the medial cutaneous and anterior cutaneous nerve of the thigh. The femoral nerve terminates as the sensory saphenous nerve of the leg. The deep branch mainly supplies muscles of the anterior compartment of the thigh, leg extensor muscles. The first motor branch innervates the iliacus. This muscle, in conjunction with the psoas major, causes medial rotation of the hip. The deep branch of the femoral nerve then descends to supply the Sartorius (the tailor's muscle). Once it passes through the femoral canal, it supplies the pectineus, a small muscle in the medial compartment of the thigh. Finally, the nerve supplies the four heads of the quadriceps femoris (vastus medialis, vastus lateralis, vastus intermedius, and rectus femoris), prime movers for leg extension at the knee joint and thigh flexion and critical for standing and stepping function. The medial and anterior cutaneous nerves of the thigh innervate the skin of the anterior thigh and the medial surface of the thigh, and saphenous nerve supplies the medial surface of the leg from the knee to the foot. The lateral femoral cutaneous nerve is a separate sensory nerve arising from L2 and L3 and supplies sensation over the lateral thigh [34, 36–38].

The obturator nerve (L2–L4) (Figure 5) passes through the large obturator foramen of the pelvis and enters the medial compartment of the thigh by passing through the obturator foramen accompanied by the obturator artery. The obturator nerve innervates the adductor muscles of the thigh, medial compartment muscles. As it goes through the foramen, it divides into anterior and posterior branches. The anterior division of the obturator nerve, lying deep to the adductor longus on the surface of the adductor brevis, gives branches to the adductor longus, the adductor brevis, and the gracilis and the skin of the medial part of the thigh. The posterior division of the obturator nerve emerges through the obturator externus after supplying it to lie on the adductor magnus. It supplies the adductor magnus and gives a branch which accompanies the femoral artery into the popliteal fossa to supply the capsule of the knee joint. The obturator nerve controls the adduction and rotation of the thigh. A small cutaneous zone on the internal thigh is supplied by a sensory fiber [34, 38].

The sacral plexus arises from the ventral rami of L4–S4 (Figure 5). The sacral plexus is situated on the posterior pelvic wall, anterior to the piriformis muscle. The ventral rami of the sacral nerves come together on the lower part of the greater sciatic foramen and unite to form a broad triangular band of nerves that innervates the lower limbs. The apex of the band is continued through the greater sciatic foramen into the gluteal region to form the sciatic nerve (L4, L5, and S1-3), the largest and longest nerve, in the body. Other branches of the sacral plexus are the superior gluteal (L4-S1), inferior gluteal (L5-S2), pudendal (S2-S4), and posterior femoral cutaneous (S2-S3) nerves. The sacral plexus also gives muscular branches to the quadratus femoris and inferior gemellus (L4-S1), obturator internus and superior gemellus (L5-S2), piriformis (S1-S2), and levator ani, coccygeus, and sphincter ani externus (S4) muscles and also contributes branches to pelvic splanchnic nerves (S2-S4) [38].

The sciatic nerve and its branch innervate all regions of the lower limb except the anterior and medial regions of the thigh [38]. The sciatic nerve leaves the pelvis by passing through the greater sciatic notch, then courses deep to the broad gluteus maximus muscle and enters to the thigh just medial to the hip joint. From there, it descends through the posterior thigh deep to the hamstrings, which it innervates. Superior to the knee joint, it branches into the tibial nerve (L4-S3), medial division, and the common fibular nerve (L4-S2), lateral division. The tibial nerve then continues posteriorly in the timeline to the calf, innervating the posterior compartment muscles of the leg (plantar flexor muscles), intrinsic foot muscles, and sensation in the sole of the foot. The common fibular nerve travels laterally and around the fibular head, dividing into the deep fibular and superficial fibular branches, which supply the muscles of the anterior (dorsiflexors) and lateral compartments (foot evertors) of the leg, respectively. The superficial fibular nerve also forms a sensory branch that supplies sensation to the anterolateral lower leg and dorsum of the foot while the deep fibular nerve supplies sensation to the web space between the first and second toes [38].

**Figure 6:**
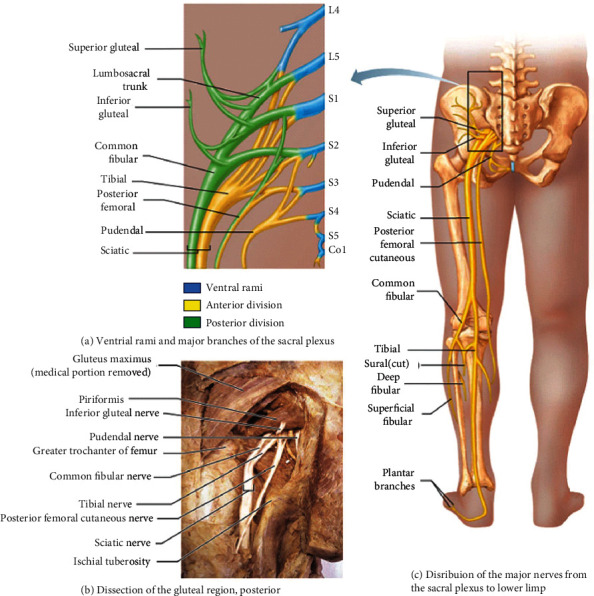
Anatomy of the sacral plexus [38].

**Figure 7:**
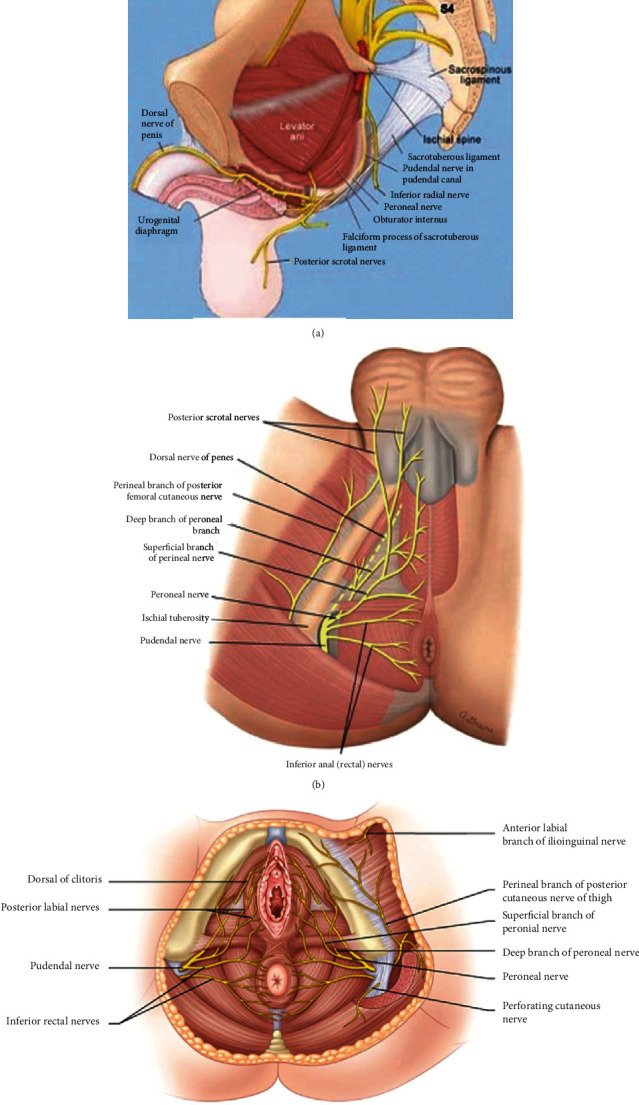
Anatomy of the pudendal nerve. (a) Origin and course of destination. (b) Pudendal nerve in the male perineum. (c) Pudendal nerve in the female perineum [1, 2].

The pudendal nerve is a mixed sacral nerve (motor 20%, sensory 50%, and autonomic 30%) [39] that provides cutaneous and muscular innervation to the majority of the perineum (Figure 6), anal canal, anus, and external male and female genetalia (scrotum, penis, mons pubis, labia majora, labia minora, clitoris, external vaginal orifice, and urethra). The pudendal nerve originates from the ventral rami of the sacral nerves (S2-S4) and then passes through the greater sciatic foramen, below the level of the piriformis (Figure 7). It passes the back of the ischial spine, between the sacrospinous and the sacrotuberous ligaments, and it enters the perineum via the lesser sciatic foramen [40–42]. The main trunk of the pudendal nerve takes an extrapelvic course superficial to the coccygeus muscle. In the upper half of the pudendal canal or within it, the pudendal nerve gives rise to the inferior rectal nerve, and at the end of the canal, it gives rise to the perineal nerve and dorsal nerves of the penis and clitoris. The inferior rectal nerve exits the pudendal canal medially and extends motor and sensory branches. Motor branches innervate the levator ani, external anal sphincter, and the cutaneous branches to perianal skin and the scrotum or labia. Perineal nerve supplies the perineum, vagina, urethra, male scrotum, labia, transverse perineal muscle, and urethral sphincter, and the dorsal nerve of the clitoris or penis supplies skin of the clitoris/penis, bulbocavernosus, and ischiocavernosus muscles [43, 44].

## 3. Clinical Presentation of Lumbar Disc Degeneration and Lumbosacral Nerve Lesion

A patient's clinical presentations and symptoms are important diagnostic tools to identify lumbar disc degeneration and lumbosacral nerve lesion. For this, the physician must conduct a physical examination and should ask many questions related to the problems [45]. Straight leg raising is the most commonly used method to diagnose lumbar disc degeneration and associated lumbosacral nerve lesions. The patient lies in the supine position, and the leg is elevated from the ankle, with the knee remaining straight. Normal patients can elevate the leg 60 to 90 degrees without pain. Patients with disc problems can only elevate the leg from 30 to 40 degrees due to produce pain. Ipsilateral straight leg rising is more sensitive, but less specific than contralateral straight leg rising. That is, nearly all patients with disc problem have pain on the straight leg raising on the affected side, but straight leg raising causes pain in many other conditions (e.g., severe hip arthritis). However, contralateral straight leg raising does not produce pain on the affected side unless the pain is due to root disease [46].

Symptoms and clinical presentations associated with lumbar disc degeneration and lumbosacral nerve lesion are discogenic pain, radical pain, muscular weakness, and cutaneous innervation defect. Discogenic pain is caused by a damaged intrinsic intervertebral disc in the lumbar region [47]. As the disc begins to degenerate, the disc itself becomes painful and movements that place stress on the disc may result in discogenic pain that comes from the disc. This is similar to any other body part injury, such as a broken bone or a cut in the skin. Discogenic pain is usually felt in the lumbar region. The pain may also feel like it is coming from the buttocks, lower thoracic, abdomen, flanks, groin, genitals, thighs, knees, calves, ankles, feet, and toes [48]. Patients with discogenic pain associated with lumbar disc degeneration may present with suddenly forced flexion and/or rotational moment, and some patients may have a spontaneous onset of symptoms. Classic discogenic pain is aggravated by activities that load the disc, such as sitting, standing, walking, flexion, rotation/twisting, lifting, vibration (e.g., riding in a car), coughing, sneezing, laughing, and the Valsalva maneuver [48].

Lesion of the lumbosacral plexus by lumbar disc degeneration leads to a lumbosacral radicular syndrome. This syndrome is characterized by a radiating pain in one or more lumbar or sacral nerve dermatomes and decreased motor function. Sometimes, it may be regarded as sciatica, ischias, or nerve root pain [49]. Radicular pain and radiculopathy are sometimes used interchangeably, although they certainly are not synonyms. In the case of radicular pain, only radiating pain is present from an inflamed or compressed nerve root. As an example, an inflamed nerve root in the lower back may radiate pain into the leg, while in the case of radiculopathy, motor loss may occurs when a compressed or inflamed nerve root results in neurological deficits, such as problems with reflexes, numbness, and/or weakness. Both syndromes frequently occur together, and radiculopathy can be a continuum of radicular pain.

Lower extremity radicular pain and radiculopathy problems due to lumbar disc degeneration are caused by compression of neural structures in the lumbosacral region [47]. Lumbar disc degeneration may compress neural structures in the lumbosacral, and this results in lumbosacral nerve roots and lumbosacral plexus lesions. Lesion of these structures results in radicular pain, weakness, numbness, or difficulty controlling specific muscles of the lower extremities, buttock, lower abdomen, and groin region. Radicular pain may be confined to a single nerve root or may involve groups of nerve roots. Pain may be of sudden or insidious onset. Radicular pain is often worsened with axial loading, sitting, standing, and bending, lifting, or twisting, and the pain feels better while walking, changing position, lying down, or even running. Numbness, tingling, weakness in the extremities, and strong pain that tends to come and go are also the features of nerve compression in the lumbosacral region [47].

Lesion of the lumbosacral plexus by lumbar disc degeneration is divided clinically into those affecting the lumbar plexus and the sacral plexus. A lumbar plexus lesion may cause symptoms in the territories of the iliohypogastric, genitofemoral, ilioinguinal, femoral, and obturator nerves [50, 51]. Patients with lumbar plexus lesion clinically present with weakness of hip flexion, knee extension, thigh adduction, and sensory loss in the lower abdomen, inguinal region, and over the entire medial, lateral, and anterior surfaces of the thigh and the medial lower leg. In lumbar plexus lesion, decrease or absence of knee jerk is common [51, 52].

Similar to lumbar plexus lesion, the sacral plexus lesion also presents with muscular weakness, loss of cutaneous sensation, and pain in the distribution areas of sacral plexus branches and gluteal nerve, sciatic nerve, tibial nerve, peroneal nerves, and pudendal nerve. In sacral plexus lesions, the muscular weakness of the lower extremities is significant. These include weakness in hip extension (gluteus maximus), hip abductors and internal rotators (gluteus medius and tensor fascia latae), knee flexion (hamstring muscles), and all muscles of the leg and foot supplied by the peroneal and tibial nerves. The diminished sensation may involve the posterior aspect of the thigh, anterolateral and posterior aspect of the leg below the knee, and almost the entire foot. The ankle jerk may be diminished or absent [34, 51].

Lesions of the sacral plexus result in weakness of the posterior thigh and muscles of the leg and feet. During sacral nerve plexus lesion, nerve fibers destined for the sciatic nerve, and the common peroneal nerve is often affected. Sciatica is defined as “pain in the distribution of the sciatic nerve due to pathology of the nerve itself.” The term “sciatica” may be confused with radicular pain as it has been used to describe any pain, including referred pain felt in the leg along the distribution of the sciatic nerve. Nevertheless, the term “sciatica” remains in common usage both in clinical practice and in publications [53, 54]. The use of the term sciatica should only be in the context of the above definitions and, as such, be distinguished from any or all other forms of pain felt in the leg, particularly referred pain [55].

Sciatica is the most common neuropathies of the lower extremities, second to common fibular neuropathy. One of the most common presentations of sciatic neuropathy is foot drop, because ankle dorsiflexion weakness, with or without lower extremity sensory impairment, may also be associated with several other clinical syndromes. Patients often experience abrupt pain radiating down the posterolateral limb, with weakness and numbness evolving more gradually [56]. In sciatic neuropathy, the clinical findings are often more consistent with injury to the common fibular division rather than tibial division, sometimes mimicking a common fibular neuropathy at the knee. This finding is particularly true of more distal lesions, as they may not affect the flexors of the knee, or of less severe sciatic nerve injury. Because the common fibular division has fewer and larger fascicles and less supportive tissue compared with the tibial division, it is thought to be more vulnerable to compression. Also, the common fibular division is tauter, and secured at the sciatic notch and fibular neck, resulting in greater potential for stretch injury [54].

Common peroneal nerve lesion is clinical characterized by weakness of foot inversion, plantar flexion, foot drop or dorsiflexor, and depressed ankle jerk [54]. Numbness and decreased sensation are also present along the anterolateral calf and dorsum of the foot [57]. Foot drop is the main feature of fibular neuropathy, and it is due to paralysis of the dorsiflexor muscles of the foot. The difficulty of eversion may be present due to peroneal muscle involvement. On examination, foot eversion is usually stronger than foot dorsiflexion. Other muscles of the posterior compartment are normal [58]. In a large study of common peroneal neuropathy, physicians clinically misdiagnosed 43% of patients as a sciatic neuropathy. This was usually because of the difficulty in assessing ankle inversion and eversion in the presence of foot drop. In sciatic neuropathy, gluteal, hamstring muscles and tibialis posterior muscles are involved [58].

Patients with pudendal nerve injury due to a sacral nerve plexus lesion typically present motor weakness of perineal muscles [59], pain, and burning sensation in the areas of the anal canal, anus, labia major, labia minor, clitoris, penis, and scrotum. Sometimes, the pain may refer to the groin, medial thigh, buttock, and abdomen. These patients may also suffer from constipation, pain, and difficulty of bowel movements, burning during urination, painful intercourse, and sexual dysfunction (uncomfortable arousal, decreased sensation, impotence, and ejaculatory dysfunction) [60, 61]. Pain due to pudendal nerve lesion is aggravated by sitting, other flexion activities of the hip (sitting, squatting, bicycling, and exercising) whereas standing or lying down relieves the discomfort [60–62].

## 4. Conclusion

During the diagnosis of patients with lumbar disc degeneration and lumbosacral nerve lesions, physicians should not use the MRI solely. It is important to assess and understand clinical presentations and pain distribution of lumbar disc degeneration and lumbosacral nerve lesions. They have to assess the patient's discogenic pain in the lumbar region, weakness of hip flexion, knee extension, and flexion, thigh adduction, ankle inversion, plantar flexion, and foot drop, perineal muscles. The patient's pain and difficulty of bowel movements, burning during urination, painful intercourse, and sexual dysfunction assessments are also critical. Besides, it is important to evaluate the loss of cutaneous sensation in the lower abdomen, inguinal region, over the medial, lateral, and anterior aspect of the thigh, the medial lower leg and in the areas of the anal canal, anus, labia major, labia minor, clitoris, penis, and scrotum. Sensory loss may also present along the posterior aspect of the thigh, anterolateral and posterior aspect of the leg below the knee, and almost the entire foot during the sacral plexus.
